# New and reemerging diseases: the importance of biomedical research.

**DOI:** 10.3201/eid0403.980308

**Published:** 1998

**Authors:** A S Fauci

**Affiliations:** National Institutes of Health, Bethesda, Maryland, USA.


### New and Reemerging Diseases: The Importance of Biomedical Research

**Anthony S. Fauci**
National Institutes of Health, Bethesda, Maryland, USA

A generation ago, it was suggested that the threat of infectious diseases would soon become an artifact of history. Today, as we approach the new millennium, the folly of this position is increasingly clear. My 87-year-old father recently reminded me of this. In the course of his lifetime, spent almost entirely in New York City, he has witnessed two pandemics of extraordinary impact: the global influenza pandemic of 1918–1919, which killed more than 20 million people worldwide, and the HIV/AIDS pandemic, which began to accelerate in the early 1980s and continues unabated in some parts of the world. In addition, at least 30 other new and reemerging diseases and syndromes have been recognized since the 1970s, including liver disease due to hepatitis C virus, Lyme disease, foodborne illness caused by *Escherichia coli* O157:H7 and *Cyclospora*, waterborne disease due to *Cryptosporidium*, hantavirus pulmonary syndrome, and human disease caused by the avian H5N1 influenza virus (Figure 1). Clearly, we remain vulnerable to new and reemerging diseases.

New diseases are superimposed on endemic diseases such as diarrheal diseases, malaria, tuberculosis (TB), and measles, which continue to exact a huge toll. Indeed, malaria and TB, among others, are reemerging in a drug-resistant form. Today, infectious diseases remain the leading cause of death worldwide and the third leading cause of death in the United States. Many pathogens are becoming increasingly resistant to standard antimicrobial drugs, making treatment difficult and in some cases impossible. Moreover, chronic conditions generally considered noninfectious actually have been found to have a microbial etiology.

### Awareness of Emerging Infections

The challenges posed by infectious diseases are recognized by the public and the media, as well as by political leaders and policy makers at the highest levels of government. There is a growing awareness that we live a global community, that diseases do not recognize borders, and that the U.S. public health community has an important role to play in fostering global health.

### The Importance of Research

The infectious diseases community faces a difficult challenge: coping with ongoing problems such as malaria and TB while preparing for the inevitable emergence of diseases that are unknown or are recognized but will reemerge in a more threatening form. Available resources must be maximized by sustaining and increasing collaboration between federal agencies, academia, industry, and nongovernmental agencies, all of which play important roles in the fight against infectious diseases.

Within the federal government, the Centers for Disease Control and Prevention's (CDC) work in detecting and tracking pathogens is critical, especially with regard to diseases that have recently emerged or have the potential for emergence. Equally important, and complementary to CDC's efforts, is basic and clinical research supported by the National Institutes of Health (NIH) and other agencies. Historically, basic research has led to important, often serendipitous, advances that have illuminated the etiology of sometimes mysterious diseases and facilitated the development of diagnostics, therapies, and vaccines (Figure 2).

At the National Institute of Allergy and Infectious Diseases (NIAID) at NIH, we have increased funding for emerging diseases from $39.3 million in fiscal year 1993 to an estimated (president's budget) $85.0 million in fiscal 1999 (Figure 3). Approximately 21% of the NIAID non-AIDS infectious diseases budget is devoted to emerging infectious diseases.

With the help of our advisory committees, we have defined five priorities in emerging and reemerging diseases research: 1) supporting the application of relevant scientific knowledge and

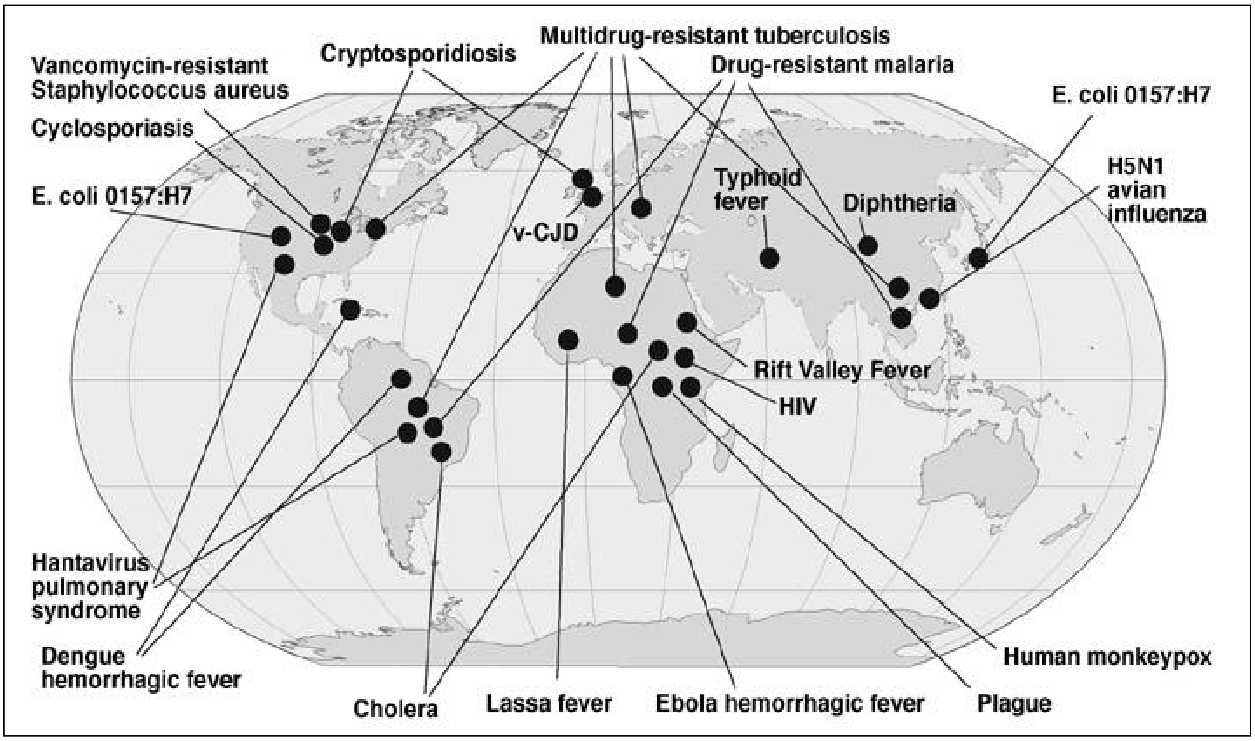

Figure 1. Examples of new and reemerging diseases.

new technologies to the detection, identification, and interdiction of emerging diseases, by expanding research on ecologic and environmental factors influencing disease emergence and transmission; 2) supporting the application of recent discoveries and new biomedical technologies to the identification, management, and control of emerging diseases, by expanding research on microbial changes and adaptations that influence disease emergence; 3) providing fundamental information for developing prevention and treatment strategies that can be employed to ameliorate disease impact, by expanding research on host susceptibility to emerging or reemerging pathogens; 4) supporting the development and validation of vaccines, therapeutics, and other control strategies for specific diseases with the potential to emerge or reemerge; and 5) strengthening the current U.S. research and training infrastructure for detecting and responding to outbreaks of infectious diseases.

Among many studies domestically and internationally, NIAID sponsors five international programs in tropical infectious diseases, most of which have components both in the United States and in the countries where the incidence of these diseases is greatest. It is essential to engage scientists in host countries and work with them collaboratively, both to tap their expertise as well as to help them build research infrastructure on their home soil.

### Successful Partnerships

The public and private sectors, including government, academia, and industry, bring complementary skills and perspectives to the research endeavor. Cross-sector collaboration can yield extraordinary dividends. A cogent example is the development of protease inhibitors for the treatment of HIV disease.

After HIV was identified in 1983, researchers funded by NIH and others began to intensively study the structural and regulatory genes of HIV and the role these genes and their products play in the replication cycle of the virus. This work led to an understanding of the importance of the HIV protease enzyme and methods to express, purify, and crystallize the enzyme. Building on these findings, researchers in the private sector designed and produced specific inhibitors of HIV protease and worked closely with the Food and Drug Administration,

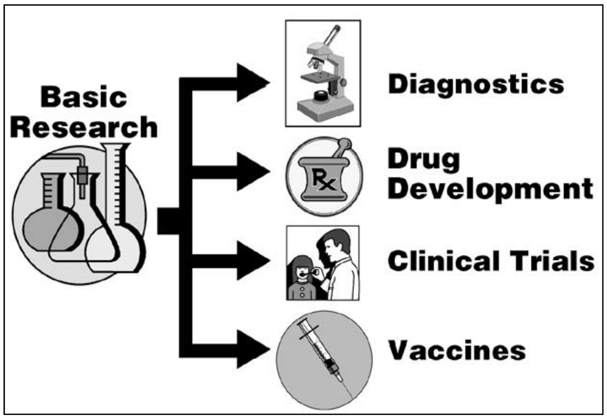

Figure 2. Emerging infectious diseases: a research approach.

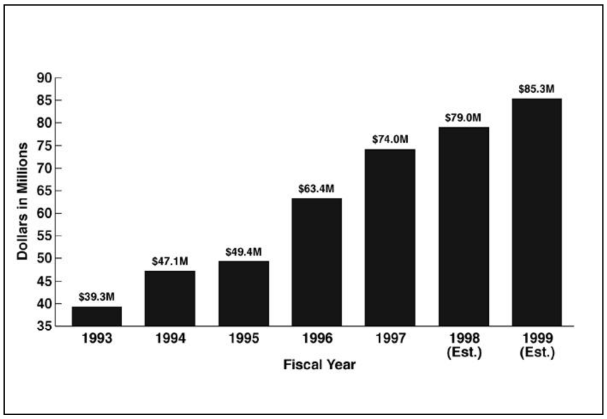

Figure 3. Emerging diseases funding (National Institute of Allergy and Infectious Diseases).

NIH, and others to assess protease inhibitors in clinical trials.

The first of four licensed protease inhibitors reached the market in December 1995. Given in combination with at least two other antiretroviral drugs, protease inhibitors dramatically reduce levels of plasma viremia in a substantial proportion of patients. Both controlled and observational studies show that these potent regimens can provide a substantial clinical benefit.

Although drug combinations that include protease inhibitors have helped many patients, it is far too soon to become complacent or declare victory. Many patients have not benefited from the new drugs or cannot tolerate their side effects, and drug resistance will inevitably become more widespread. The development of the next generation of antiretroviral agents is crucial and will require the skills of investigators in both the public and private sectors. However, the cost of antiretroviral drugs will probably keep them beyond the reach of much of the developing world; therefore, the development of an HIV vaccine is of paramount importance.

### Malaria Initiatives at NIH

Until relatively recently, AIDS was virtually the only emerging disease with global impact that was widely discussed in the United States; however, other diseases such as malaria and TB have actually caused more illnesses and deaths over the past 2 decades.

Malaria kills up to three million persons each year, most of them children in sub-Saharan Africa. In the past year, NIH has worked with research organizations and donor agencies from around the world to form a coalition called the Multilateral Initiative on Malaria. This unprecedented initiative will enhance international collaborations, encourage the involvement in malaria research of scientists from malaria-endemic countries, and identify additional malaria research resources. In addition, NIH has bolstered its long-term commitment to malaria research. NIH-supported malaria projects—many in collaboration with other government and international agencies—include 1) a new repository of materials available to researchers worldwide; 2) basic, field-based, and clinical research on all phases of malaria research; and 3) projects to determine the genetic sequences of important malaria species.

### Responding to Avian H5N1 Influenza

An outbreak of avian H5N1 influenza in Hong Kong recently alarmed the medical community and the world. The multinational response to this outbreak has involved the close collaboration of many organizations (Figure 4). As part of NIH's long-standing research into respiratory viruses, we had in our reagent repository the specific antisera needed to quickly develop test kits that were used effectively by CDC and others for detecting and tracking the virus. We also have supported the rapid production of a recombinant vaccine against avian influenza virus for use in laboratory and health-care personnel at risk. Without a strong research base, the rapid response to this emergency would not have been possible.

### Vaccine Development

With avian flu, malaria, AIDS, and other new and reemerging diseases, an important goal of NIH is the development of vaccines. If just four recently developed vaccines (hepatitis B, rotavirus, *Haemophilus influenzae* type b, and acellular pertussis) were universally administered, more than three million deaths could be prevented each year.

Historically, scientific advances in microbiology and related disciplines have driven the development of new vaccines. For example, the identification of microbial toxins, as well as methods to inactivate them, allowed the development of some of our earliest vaccines, including those for diphtheria and tetanus. In the 1950s, new tissue culture techniques ushered in a new generation of vaccines, including measles, mumps, and rubella. In recent years we have seen rapid advances in our understanding of the immune system and host-pathogen interactions, as well as technical advances such as recombinant DNA technology, peptide synthesis, and gene sequencing. Each of these has facilitated the development of new vaccines and vaccine candidates for important pathogens.

Sequence information can be used in many ways and promises to be useful in identifying antigens to incorporate into vaccines, as well as determining the factors that influence the antigenicity or virulence of a microbe. The complete genetic sequences of more than 13 microorganisms have now been published. More than 60 other sequencing projects for medically important pathogens, such as *Plasmodium* spp., *Mycobacterium* spp., *Chlamydia trachomatis*, *Vibrio cholerae,* and *Neisseria gonorrhoeae,* are under way.

### Conclusion

The importance of basic research to the control of emerging and reemerging diseases cannot be overemphasized. Emerging diseases research encompasses many disciplines, and research advances that fall under the rubric of emerging diseases will be relevant not only to specific diseases being studied but to a broad range of disciplines such as vaccinology, immunology, and drug development (Figure 5). In turn, research in these areas is critical to advances in emerging and reemerging diseases. With a sustained commitment to basic research and cross-sector collaboration, important scientific findings and technological advances can be translated into improved global health and reduced susceptibility to new microbial threats.

### Acknowledgment

The author thanks Greg Folkers for helpful discussion related to the preparation of this manuscript.

### References

1. Institute of Medicine, Board on International Health. America's vital interest in global health. Washington: National Academy Press; 1997.
2. Centers for Disease Control and Prevention. *Staphylococcus aureus* with reduced susceptibility to vancomycin—United States, 1997. MMWR Morb Mortal Wkly Rep 1997;46:765-6.

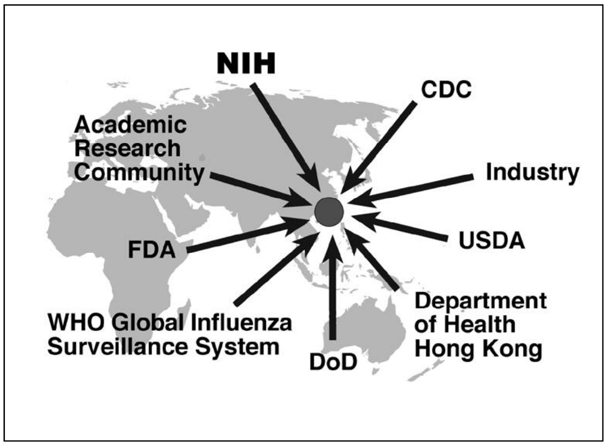

Figure 4. Response to H5N1 avian influenza outbreak in Hong Kong.

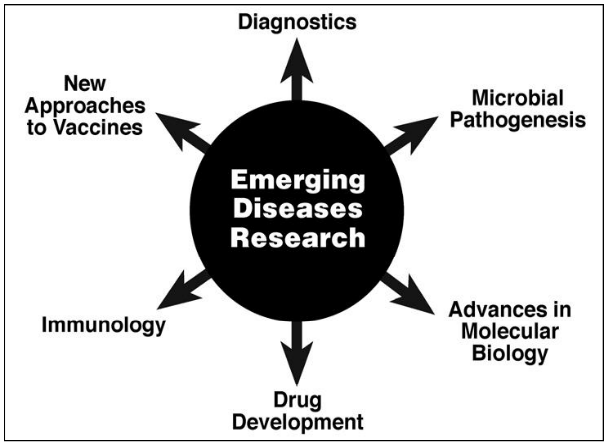

Figure 5. Benefits of emerging diseases research.

3. Centers for Disease Control and Prevention. Update: isolation of avian influenza A (H5N1) viruses from humans—Hong Kong, 1997-1998. MMWR Morb Mortal Wkly Rep 1998;26:1245-7.
4. The CVI strategic plan: managing opportunity and change: a vision of vaccination for the 21st century. Geneva: Children's Vaccine Initiative, 1997. Sponsored by UNICEF, United Nations Development Program, World Health Organization, World Bank, Rockefeller Foundation.
5. Two cheers for the multilateral malaria initiative. [editorial]. Nature 1997;388:211.
6. Fauci AS. Biomedical research in an era of unlimited aspirations and limited resources. Lancet 1996;348:1002-3.
7. The Institute for Genomic Research. TIGR Microbial Database [database online] [cited 1998 Apr 1]. Available from: URL: http://www.tigr.org.
8. World Health Organization. World Health Report 1997—conquering suffering, enriching humanity. Geneva: The Organization; 1997.
